# Identification of snoRNA SNORA71A as a Novel Biomarker in Prognosis of Hepatocellular Carcinoma

**DOI:** 10.1155/2020/8879944

**Published:** 2020-09-26

**Authors:** Yuan Ding, Zhongquan Sun, Sitong Zhang, Liuzhi Zhou, Qianhui Xu, Dongkai Zhou, Yanjie Li, Xin Han, Hao Xu, Yang Bai, Chang Xu, Hao Ding, Yao Ge, Weilin Wang

**Affiliations:** ^1^Department of Hepatobiliary and Pancreatic Surgery, The Second Affiliated Hospital, Zhejiang University School of Medicine, Hangzhou 310009, China; ^2^Key Laboratory of Precision Diagnosis and Treatment for Hepatobiliary and Pancreatic Tumor of Zhejiang Province, Hangzhou 310009, China; ^3^Research Center of Diagnosis and Treatment Technology for Hepatocellular Carcinoma of Zhejiang Province, Hangzhou 310009, China; ^4^Clinical Medicine Innovation Center of Precision Diagnosis and Treatment for Hepatobiliary and Pancreatic Disease of Zhejiang University, Hangzhou 310009, China; ^5^Clinical Research Center of Hepatobiliary and Pancreatic Diseases of Zhejiang Province, Hangzhou 310009, China

## Abstract

**Background:**

Small nucleolar RNAs (snoRNAs) have been proved to play important roles in various cellular physiological process. Recently, dysregulation of snoRNA SNORA71A has been found involved in tumorigenesis of various malignant cancers. However, the emerging effects of SNORA71A in hepatocellular carcinoma (HCC) remain largely unclear. In this study, we aimed to explore the SNORA71A expression and its underlying significance in HCC.

**Methods:**

Expression of SNORA71A in cell lines and clinical specimens was measured by quantitative real-time PCR. Then, all enrolled HCC patients were divided into low and high SNORA71A expression subgroups and then they were compared in the aspects of clinical features as well as survival outcome by respective statistical analysis methods.

**Results:**

SNORA71A was significantly downexpressed in SK-HEP-1 (*P* = 0.001), Huh-7 (*P* < 0.001), Hep3B (*P* < 0.001), and clinical HCC specimens (*P* = 0.006). Comparing the clinical features between SNORA71A expression subgroups, it showed that low SNORA71A expression was significantly associated with large tumor diameter, multiple lesions, capsular invasion, bad tumor differentiation, and TNM stage (*P* < 0.05). Furthermore, it was found that HCC patients with lower SNORA71A expression had higher risk in postoperative tumor relapse (median time: 9.5 vs. 35.2 months; low vs. high; *P* < 0.001) and poor overall survival (median time: 36.8 vs. 52.9 months; low vs. high; *P* < 0.001). Besides, SNORA71A expression served as independent risk factors for tumor-free (HR = 0.450; 95% CI [0.263-0.770]; *P* = 0.004) and long-term survival (HR = 0.289; 95% CI [0.127-0.657]; *P* = 0.003).

**Conclusions:**

Our study for the first time demonstrated that downregulation of SNORA71A could serve as a novel biomarker for clinical assessment and prognostic prediction of HCC patients.

## 1. Introduction

Hepatocellular carcinoma (HCC) is the fifth most common cancer and causes the fourth most cancer-related deaths in the world [1]. There are a series of risk factors to induce the tumorigenesis of HCC, mainly including chronic hepatitis virus infection, fatty diabetes, excessive alcohol intake, and nonalcoholic fatty liver disease [2]. Despite great achievements in the treatment of hepatocellular carcinoma with respect to liver resection and chemotherapy, the survival outcomes of HCC patients remain dissatisfactory for its high incidence of relapse, metastasis, and mortality [3]. Even as the most commonly and important means for HCC diagnosis, serum AFP monitoring is not sufficient to predict the postoperative survival for HCC patients effectively [4]. Therefore, it is vital and significant to identify a novel reliable clinical marker for HCC prognostication.

Small nucleolar RNA (snoRNA) is a type of noncoding RNA widely found in the nucleoli of eukaryotic cells [5]. It has a length of 0-300 nt and has conserved structural elements. Although these snoRNAs were once considered with single function and limited effects in pre-rRNA processing, recent accumulating evidence showed that some snoRNAs also participated in various cellular physiological processes, including cell proliferation, differentiation, epigenetic, regulation. And some dysregulation of snoRNA was proved to serve as tumor suppressor genes or oncogenes in various cancers [6–9]. A review from Han et al. clearly stated that SNHG1, SNHG6, SNHG15, SNHG16, and SNHG20 can play varied roles in HCC through different regulatory mechanisms. These SNHGs can promote and inhibit tumorigenesis [10]. For instance, snoRNA SNHG1 promoted tumorigenesis and progression of colorectal cancer via negative regulation of miR-137 [11]. Besides, snoRNA SNORA18 acted as a tumor suppressor gene in hepatocellular carcinoma [12].

SNORA71A, encoded by the third intron of snoRNA host gene 17 (SNHG17) cloning, commonly guided the pseudouridine of U406 in 18S rRNA [13]. In recent, more and more studies reported that SNORA71A was dysregulated in various types of cancers, such as lung cancer [14] and breast cancer [15], and played crucial roles in tumor progression. However, the emerging effects and potential mechanisms of SNORA71A in hepatocellular carcinoma remain largely unclear. In this study, we aimed to identify the SNORA71A expression features in HCC and its underlying clinical significance.

## 2. Materials and Methods

### 2.1. Patients and Tissue Samples

To reduce the potential confounding factors, none of the enrolled patients received any preoperative radiotherapy, chemotherapy, or endocrine therapy. All enrolled patients were diagnosed with HCC by histopathological examination. All patients involved in the study were written informed consent and approval. This study was approved by the Institutional Ethics Committee of the First Affiliated Hospital of Zhejiang University (Hangzhou, China).

HCC tissues and adjacent liver tissues were received from 132 consecutive HCC patients, who underwent liver resection between January 2013 and December 2014. HCC tissue specimens underwent 2 pathological examinations by pathologists for TNM stage. Tissues were immediately snap frozen into liquid nitrogen postoperation and then stored at -80°C for RNA isolation. Patient data was collected from the database of the hospital electronic system, including individual information, liver function indexes, alpha fetal protein value, HBV infection, lesion features, pathological differentiation, and TNM classification.

### 2.2. Follow-Up of Tumor-Free and Long-Term Survival

After hepatectomy, all patients were followed up for 5 years. After the initial surgery, the time terminal of tumor-free survival included the recurrence of HCC, HCC distant metastasis, or death from any cause without cancer-related events, while long-term survival was calculated until death or the last follow-up time in 5 years. Death of patients was ascertained by the family members and the review of public hospital records. All staff who collected all the following-up data of enrolled patients were blind to participant status.

### 2.3. Cell Culture

Human normal hepatocyte cell line (QSG-7701) and HCC cell lines (SK-HEP-1, Huh-7, and Hep3B) obtained from the laboratory were maintained in the DMEM medium (BI, Israel) with 10% FBS (BI, Israel) as well as 100 U/ml penicillin and 100 mg/ml streptomycin (Sangon, China) at 37°C in a humidified incubator (Thermo Fisher, USA) containing 5% CO_2_.

### 2.4. RNA Extraction and Quantitative Real-Time PCR

Total RNA from cell lines and tissue specimens was extracted using Trizol reagent (Invitrogen, USA) according to the manufacturer's instructions. RNA was reversely transcribed into cDNA using cDNA Reverse Transcription Kit (Vazyme, Nanjing, China). To determine the expression level of SNORA71A, cDNA sample was tested with quantitative real-time polymerase chain reaction (RT-PCR) (Roche, Basel, Switzerland). All samples were tested in triplicate. The expression of glyceraldehyde-3-phosphate dehydrogenase (GAPDH) was used as an endogenous control, and the relative expression of SNORA71A was calculated by comparative Ct method formula 2^−ΔΔCt^. The sequences of all PCR primers used were as follows (5′-3′): GAPDH: CAGGAGGCATTGCTGATGAT (forward), GAAGGCTGGGGCTCATTT (reverse); SNORA71A: AGGTCATTGATAGTGCAGGGAG (forward), GGTTCGGATGGGATAGGGT (reverse).

### 2.5. Statistical Analysis

All statistical analyses were performed using the SPSS (Statistical Package for the Social Sciences) 19.0 (SPSS, Chicago, IL). The differences between two independent groups were analyzed using Student's *t*-test. The correlations between SNORA71A expression and different clinical characteristics were performed with chi-squared test. The differences of tumor-free and long-term survival in different subgroups were assessed by Kaplan–Meier survival plots and log-rank tests. Associations between HCC prognosis and clinical parameters were first performed with univariate analysis and then gradually analyzed using multiple logistic regression for those significantly differing factors. All data were expressed with mean ± standard deviation (SD), and *P* < 0.05 was recognized as statistically significant difference.

## 3. Results

### 3.1. The Expression Levels of SNORA71A in HCC

**Figure 1:**
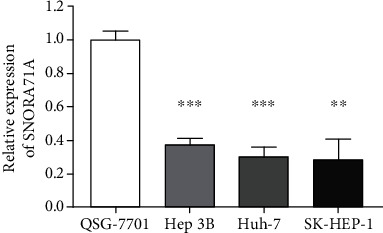
Comparison of SNORA71A expression in cell lines. Compared to normal liver cell line QSG-7701, SNORA71A was significantly suppressed in HCC cell lines (SK-HEP-1, *P* = 0.001; Huh-7, *P* < 0.001; Hep3B, *P* < 0.001). ^∗∗^*P* < 0.01 and ^∗∗∗^*P* < 0.001.

**Figure 2:**
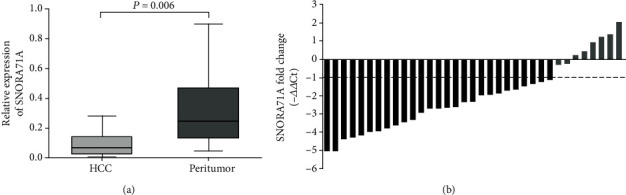
Expression patterns of SNORA71A in HCC tissue specimens. (a) Compared to corresponding adjacent liver tissues, SNORA71A expression was significantly lower in HCC tissues (*P* < 0.01). (b) Waterfall plot showed that SNORA71A was downregulated by at least twofold in 77.2% of paired HCC tissues. △△Ct = (Ct SNORA71A–Ct GAPDH) of HCC lesions − (Ct SNORA71A–Ct GAPDH) of adjacent liver tissue.

To determine whether SNORA71A was differentially expressed in HCC, firstly human normal hepatocyte (QSG-7701) and HCC cell lines (SK-HEP-1, Huh-7, and Hep3B) were used to analyze SNORA71A expression by quantitative RT-PCR. As shown in Figure 1, the SNORA71A expression in SK-HEP-1 (*P* = 0.001), Huh-7 (*P* < 0.001), and Hep3B (*P* < 0.001) were remarkably lower than QSG-7701, respectively. Then, as shown in Figure 2, the similar results were also found in clinical tissues' examination that SNORA71A expression was mostly downexpressed in HCC tissues compared with adjacent liver tissues (*P* = 0.006). Therefore, these results suggested that SNORA71A might serve as a tumor suppressive role in HCC.

### 3.2. Relationships of SNORA71A Expression with Clinicopathologic Characteristics of HCC Patients

According to individual SNORA71A expression, all 132 HCC patients were divided into high and low expression subgroups. The relationships of SNORA71A expression with clinicopathologic characteristics of HCC patients were statistically analyzed. In Table 1, low SNORA71A expression was significantly associated with large tumor diameter (*P* = 0.016), multiple lesions (*P* = 0.017), capsular invasion (*P* = 0.015), bad tumor differentiation (*P* = 0.027), and TNM stage (*P* = 0.004). However, there was no statistical significance between SNORA71A expression with other clinicopathologic characteristics, such as hepatitis B, AFP, or cirrhosis.

### 3.3. Downregulation of SNORA71A Predicts Poor Prognosis in HCC Patients

**Figure 3:**
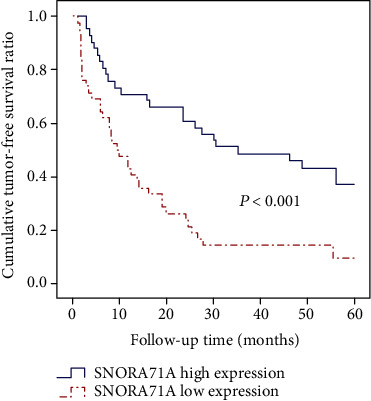
Cumulative tumor-free survival curves of patients in low and high SNORA71A expression subgroups.

**Figure 4:**
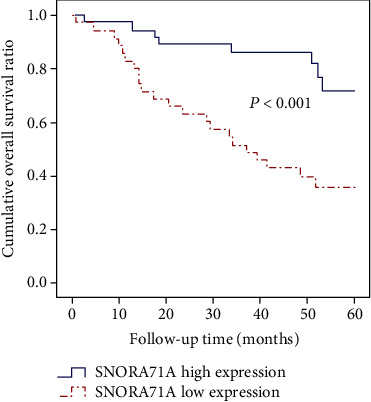
Cumulative overall survival curves of patients in low and high SNORA71A expression subgroups.

To identify whether SNORA71A expression could serve as a novel biomarker to predict prognosis of HCC patients, the tumor-free and long-term survival data of two SNORD31 expression subgroups were compared using Kaplan–Meier method. According to the results from Figures 3 and 4, we found that HCC patients with high SNORA71A expression tended to present with lower tumor recurrence rate after hepatectomy and better overall survival condition. Between the subgroups with different SNORA71A expression levels, the median tumor-free survival time (9.5 vs. 35.2 months; low vs. high; *P* <0.001) and median long-term survival time (36.8 vs. 52.9 months; low vs high; *P* <0.001) were significantly different.

### 3.4. Identification of Independent Prognostic Factors for HCC Prognostication

To determine the independent risk factors for HCC patient's survival, various clinicopathological factors and SNORA71A expression were analyzed in univariate and multivariate Cox regression analysis. In Tables 2 and 3, SNORA71A expression was proved to independently relate with tumor-free (HR [95% CI]: 0.450 [0.263-0.770]; *P* = 0.004) as well as long-term survival (HR [95% CI]: 0.289 [0.127-0.657]; *P* = 0.003) of HCC patients. These results supported that SNORA71A expression was capable of serving as the biomarker for prognosis assessment in HCC patients.

## 4. Discussion

Hepatocellular carcinoma is the most common type of liver cancer and solid tumors with fast progress and poor prognosis [16]. For HCC commonly presented with occult progression in vivo, multiple HCC patients are diagnosed at middle or even late stages [17]. Although several clinical characteristics have listed into the criteria for predicting the prognosis of HCC patients, such as tumor diameters, TNM stage, and AFP, these classification schemes are limited to become common predictors for HCC patients due to dissatisfactory sensitivity and specificity [18]. In the past 40 years, AFP has been used for monitoring the HCC, however, with poor sensitivity and specificity: 39% to 65% and 76% to 94% [19, 20]. Moreover, Zhang and Yang's study indicated that AFP level rarely rose when HCC tumor mass was less than 2 cm in diameter [21]. Therefore, it is important and necessary to explore a novel biomarker in HCC patients.

As one kind of noncoding RNA, small nucleolar RNA (snoRNA) is originally found to make function in regulating the pseudouridine of rRNA [22]. Nowadays, emerging evidence showed that snoRNA also played important regulatory roles in tumorigenesis of various cancers, including liver cancer and breast cancer [23–25]. Among that, Cui et al. indicated that upregulation of snoRNA SNORA23 promoted invasion and metastasis of xenograft tumors in mice by modulating the spectrum repeat-containing nuclear envelope 2 [26]. Baral et al. reported that SNORD126, SNORD78, ACA11, SNORA47, and SNORD76 may also serve as an important prognostic marker related to clinical features. Abnormal expression of snoRNA promotes cell proliferation and leads to the development of liver cancer [27]. Besides, Cao's team reported for the first time that dysregulation of snoRNA SNORA18L5 could increase the risk of HBV-associated hepatocellular carcinoma for abnormity of ribosomal RNA maturation [28]. In HCC, moreover, snoRNA SNORD113-1 was also found to be significantly downregulated and functions as a tumor suppressor role [23]. SNHG16, SNORD76, and SnoU2_19 can regulate the development of HCC through Wnt/*β*-catenin signaling pathway [29]. Therefore, these indicated that some specific snoRNAs had underlying abilities to serve as biomarker for clinical evaluation of HCC patients.

In our study, we for the first time analyzed the expression level of SNORA71A in both HCC cell lines and tissues and elucidated that SNORA71A was universally downregulated in HCC. Moreover, a total of 132 patients' analyses proved that SNORA71A expression was correlated with tumor diameter, multiple lesions, capsular invasion, differentiation, and TNM stage. And our results showed that SNORA71A expression was an independent risk factors for both tumor-free survival and long-term survival of HCC patients.

In conclusion, our research for the first time demonstrated that SNORA71A could serve as a novel biomarker for prognostication and therapeutic monitoring for HCC patients. And more studies are needed to explore the specific mechanisms and verify our findings in the future.

## Figures and Tables

**Table 1:** Correlation between SNORA71A expression and clinicopathological features in HCC patients.

Characteristics	Total	SNORA71A expression		*P* value
		Low (*n* = 66)	High (*n* = 66)	
Age, ys				0.138
<60	85	46 (0.697)	39 (0.591)	
≥60	47	20 (0.303)	27 (0.409)	
Gender				0.212
Female	16	6 (0.091)	10 (0.152)	
Male	116	60 (0.909)	56 (0.848)	
Hepatitis B				0.310
Positive	113	58 (0.879)	11 (0.167)	
Negative	19	8 (0.121)	55 (0.833)	
Cirrhosis				0.361
Present	79	38 (0.576)	41 (0.621)	
Absent	53	28 (0.424)	25 (0.379)	
AFP (ng/l)				0.429
<400	82	42 (0.636)	40 (0.606)	
≥400	50	24 (0.364)	26 (0.394)	
Tumor diameter				0.005^∗^
<5 cm	34	10 (0.152)	24 (0.364)	
≥5 cm	98	56 (0.848)	42 (0.636)	
Multiple lesions				0.017^∗^
Present	22	16 (0.242)	6 (0.091)	
Absent	110	50 (0.758)	60 (0.909)	
Vessel carcinoma embolus				0.160
Present	34	20 (0.303)	14 (0.212)	
Absent	98	46 (0.697)	52 (0.788)	
Microvascular invasion				0.500
Present	7	4 (0.061)	3 (0.045)	
Absent	125	62 (0.939)	63 (0.955)	
Capsular invasion				0.015^∗^
Present	49	31 (0.470)	18 (0.273)	
Absent	83	35 (0.530)	48 (0.727)	
Differentiation				0.027^∗^
Low	70	41 (0.621)	29 (0.439)	
High/moderate	62	25 (0.379)	37 (0.561)	
TNM stage				0.004^∗^
I~II	91	38 (0.576)	53 (0.803)	
III~IV	41	28 (0.424)	13 (0.197)	

AFP = alpha fetal protein; TNM = tumor-node-metastasis; ^∗^*P* < 0.05; values are mean ± standard deviation or *n* (%).

**Table 2:** Univariate and multivariate analysis of tumor-free survival in HCC patients.

Clinicopathologic parameters	Univariate analysis		Multivariate analysis	
	HR (95% CI)	*P*	HR (95% CI)	*P*
Age (<60 vs. ≥60)	0.734 (0.432-1.246)	0.252		
Gender (female vs. male)	1.397 (0.559-3.490)	0.474		
Hepatitis B (negative vs. positive)	1.608 (0.763-3.386)	0.212		
AFP (<400 vs. ≥400)	1.033 (0.618-1.725)	0.901		
Cirrhosis (present vs. absent)	1.172 (0.698-1.968)	0.549		
Microvascular invasion (present vs. absent)	1.272 (0.460-3.520)	0.643		
Tumor differentiation (low vs. high/moderate)	0.760 (0.457-1.262)	0.289		
Capsular invasion (present vs. absent)	1.521 (0.904-2.560)	0.114		
Multiple lesions (present vs. absent)	1.783 (0.980-3.242)	0.058	1.189 (0.546-2.593)	0.663
Vessel carcinoma embolus (present vs. absent)	1.699 (0.957-3.016)	0.071	1.384 (0.771-2.485)	0.276
TNM stage (I~II vs. III~IV)	2.006 (1.195-3.369)	0.008	1.577 (0.922-2.699)	0.097
Tumor diameter (<5 cm vs. ≥5 cm)	2.604 (1.402-4.839)	0.002	2.154 (1.140-4.070)	0.018^∗^
SNORA71A expression (low vs. high)	0.385 (0.228-0.650)	<0.001	0.450 (0.263-0.770)	0.004^∗^

AFP = alpha fetal protein; TNM = tumor-node-metastasis; HR = hazard ratio; ^∗^*P* < 0.05 was considered statistically significant.

**Table 3:** Univariate and multivariate analysis of overall survival in HCC patients.

Clinicopathologic parameters	Univariate analysis		Multivariate analysis	
	HR (95% CI)	*P*	HR (95% CI)	*P*
Age (<60 vs. ≥60)	1.067 (0.514-2.216)	0.861		
Gender (female vs. male)	0.541 (0.231-1.266)	0.157		
Hepatitis B (negative vs. positive)	0.895 (0.365-2.191)	0.808		
AFP (<400 vs. ≥400)	1.398 (0.682-2.866)	0.360		
Cirrhosis (present vs. absent)	1.208 (0.575-2.540)	0.618		
Microvascular invasion (present vs. absent)	1.791 (0.540-5.938)	0.341		
Tumor differentiation (low vs. high/moderate)	0.876 (0.424-1.809)	0.720		
Capsular invasion (present vs. absent)	2.175 (1.063-4.452)	0.033	1.436 (0.662-3.116)	0.360
Tumor diameter (<5 cm vs. ≥5 cm)	3.480 (1.210-10.005)	0.021	1.952 (0.620-6.143)	0.253
Multiple lesions (present vs. absent)	2.410 (1.140-5.095)	0.021	1.125 (0.429-2.952)	0.810
Vessel carcinoma embolus (present vs. absent)	2.075 (0.949-4.538)	0.068	2.047 (0.898-4.665)	0.088
TNM stage (I~II vs. III~IV)	3.061 (1.480-6.331)	0.003	2.637 (1.263-5.507)	0.010^∗^
SNORA71A expression (low vs. high)	0.257 (0.114-0.580)	0.001	0.289 (0.127-0.657)	0.003^∗^

AFP = alpha fetal protein; TNM = tumor-node-metastasis; HR = hazard ratio; ^∗^*P* < 0.05 was considered statistically significant.

## Data Availability

The datasets used and/or analyzed during the current study are available from the corresponding author on reasonable request.

## Abbreviations

HCC: Hepatocellular carcinoma
snoRNA: Small nucleolar RNA
AFP: Alpha fetal protein
TNM: Tumor-node-metastasis
HR: Hazard ratio.
